# Chemical Entity Semantic Specification: Knowledge representation for efficient semantic cheminformatics and facile data integration

**DOI:** 10.1186/1758-2946-3-20

**Published:** 2011-05-19

**Authors:** Leonid L Chepelev, Michel Dumontier

**Affiliations:** 1 Department of Biology, Carleton University, Ottawa, Canada; 2 Institute of Biochemistry, Carleton University, Ottawa, Canada; 3 School of Computer Science, Carleton University, Ottawa, Canada

## Abstract

**Background:**

Over the past several centuries, chemistry has permeated virtually every facet of human lifestyle, enriching fields as diverse as medicine, agriculture, manufacturing, warfare, and electronics, among numerous others. Unfortunately, application-specific, incompatible chemical information formats and representation strategies have emerged as a result of such diverse adoption of chemistry. Although a number of efforts have been dedicated to unifying the computational representation of chemical information, disparities between the various chemical databases still persist and stand in the way of cross-domain, interdisciplinary investigations. Through a common syntax and formal semantics, Semantic Web technology offers the ability to accurately represent, integrate, reason about and query across diverse chemical information.

**Results:**

Here we specify and implement the Chemical Entity Semantic Specification (CHESS) for the representation of polyatomic chemical entities, their substructures, bonds, atoms, and reactions using Semantic Web technologies. CHESS provides means to capture aspects of their corresponding chemical descriptors, connectivity, functional composition, and geometric structure while specifying mechanisms for data provenance. We demonstrate that using our readily extensible specification, it is possible to efficiently integrate multiple disparate chemical data sources, while retaining appropriate correspondence of chemical descriptors, with very little additional effort. We demonstrate the impact of some of our representational decisions on the performance of chemically-aware knowledgebase searching and rudimentary reaction candidate selection. Finally, we provide access to the tools necessary to carry out chemical entity encoding in CHESS, along with a sample knowledgebase.

**Conclusions:**

By harnessing the power of Semantic Web technologies with CHESS, it is possible to provide a means of facile cross-domain chemical knowledge integration with full preservation of data correspondence and provenance. Our representation builds on existing cheminformatics technologies and, by the virtue of RDF specification, remains flexible and amenable to application- and domain-specific annotations without compromising chemical data integration. We conclude that the adoption of a consistent and semantically-enabled chemical specification is imperative for surviving the coming chemical data deluge and supporting systems science research.

## Background

The importance of cataloguing and adequately representing chemical information has been realized fairly early in the development of chemistry and related sciences. From the dawn of the era of organic synthesis, thousands of chemical entities, reactions, and experimental outcomes were catalogued and stored in a human-readable form, some dating to as early as the eighteenth century when the understanding of molecular reactivity and chemical structure was nowhere near its current level (preserved in e.g. [1]). During the relatively long history of the development of chemical information archiving technologies, a large number of persistent redundancies and factors complicating chemical knowledge federation have been introduced. It may be argued, however, that these problems may be reduced to three major categories, some of which have been only recently partially addressed: i) a lack of a consensus canonical identifiers of all chemical entities, including reactions and macromolecules, as well as and their constituents, ii) absence of a single common flexible representation to satisfy the needs of most sub-disciplines of chemistry, and iii) a lack of a consensus chemical database structure or schema. We argue that the bulk of present-day complications in integrating chemical information can be traced to these three problems and that until an information representation that addresses these issues is introduced, truly integrative chemical research shall be a complicated and costly endeavour.

### Consensus Chemical Entity Identifiers

Many modern chemical databases rely on internal chemical entity identifiers which are usually created in a sequential manner, producing an index whose value increases for every new chemical entity in the database [2-5]. Unfortunately, such indexing systems require manual or semi-automated cross-database matching, resulting in difficulties of federating chemical data. Attempts to create reproducible and canonical molecular identifiers that bear molecular graph information could be dated to the introduction of computers to the field of chemistry in the 20^th ^century. The fact that molecules could be represented as graphs and features of these graphs could be used to arrive at a shorthand depiction of molecular structure, had swiftly led to the creation of the first fragment-based line notation in chemistry: the Wiswesser Line Notation (WLN) [6]. In this notation, molecular fragments were abbreviated and recorded with a limited character set to reconstitute various molecular parts and their connectivity. Unfortunately, no efficient way to create a *canonical *molecular representation for this line notation existed, meaning that a given molecule could be referred to by multiple different WLN strings in different chemical databases. This shortcoming was overcome with the introduction of the SMILES notation [7], which explicitly represented chemical molecules as graphs with atoms being nodes and bonds edges, along with an efficient algorithm to create a canonical, reproducible SMILES string representation of a given molecule. Unfortunately, multiple algorithms for SMILES canonicalization have been devised over the years, leading to software-, and therefore, database-specific canonical molecular SMILES representations. Finally, The International Chemical Identifier (InChI) notation has addressed this issue by providing algorithms and software to enable consistent canonical representation of chemical structures, but has unfortunately not yet addressed the efficient canonical representation of many other chemical entities, such as reactions and macromolecules [8]. More recently, due to the unwieldy nature of InChI for many larger molecules as well as web search engine complications, InChI keys have been introduced, producing a 25-character hash based on the elements of chemical graph structure [8]. Although InChI keys cannot be used to reconstitute chemical structure without lookup tables, their use has enabled cross-database chemical searches using common web search engines. It is not unreasonable to believe that a universal adoption of the IUPAC standard InChI keys in the role of database indexes could potentially facilitate knowledge federation immensely.

### Common Chemical Information Representation

Simple line notations have been useful as chemical structure identifiers and bearers of information necessary for the vast majority of cheminformatics tasks, such as chemical database searching or basic reactive transformation outcome predictions. However, these notations could not address the needs of structural, biological, and computational chemists, among others. For this purpose, myriads of chemical file formats incorporating elements of discipline-specific controlled annotations and geometric molecular configuration, have been developed over the past half century. One of the most popular formats to address this need has been the Structure-Data File (SDF) [9], which combines molecular structural and atomic connectivity information with data annotations. Unfortunately, these annotations may often be confusing or contradictory, as they commonly bear no units, data source information, or specific references to the moieties or molecular entities to which the annotations correspond. For instance, an octanol-water partition coefficient annotation may be specified as follows.

> <logP>

1.3856

While it may be a straightforward annotation to the creators of a given database, and while it may be more or less easily interpreted by a human agent, it bears no information with respect to corresponding units, algorithms, or parameters used in generating this value. Furthermore, if two different databases containing SDF data for partition coefficients for the same molecule were to be integrated, this integration would require human interpretation, specialized parsers, and if a relational database is used, a special field to store this information in order to enable queries over it. This task is convoluted by the limited availability of annotation specification or outright lack thereof, prompting many cheminformatics applications to re-evaluate descriptor values in a given study.

Thus, an ideal representation would be able to refer to every chemical entity and its part unambiguously and to capture information in a controlled, reproducible, and machine-understandable way to enable machine reasoning and to facilitate data integration. To address this, numerous XML-based chemical representation schemes have been created (e.g. [10-12]), enabling highly detailed reaction modeling and chemical representations but unfortunately they have neither been widely embraced by the chemical community, nor have they allowed for seamless machine-mediated information integration. One XML-based representation, the Chemical Markup Language [13], backed by a controlled vocabulary, has been rather successful in specifying most aspects of chemistry, from small molecules and their connectivity to polymers and crystal structures [14].

**Figure 1:**
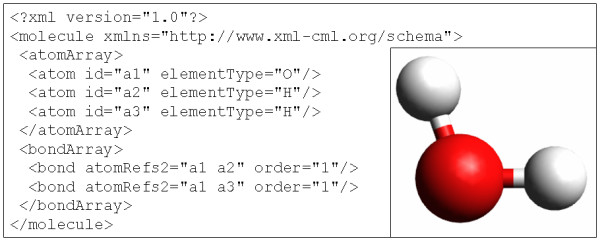
**A simplified specification of a water molecule in CML**.

Unfortunately, while most elements of this specification can be parsed out using one of the many XML libraries, certain elements do not render themselves to facile interpretation. Consider the sample CML specification of a water molecule (Figure 1). In order to identify the member atoms in a given bond, it is necessary to carry out string processing as an intermediate step. Further, while many of the elements of CML are defined in a controlled vocabulary, the lack of explicit, consistent, and formal axiomatization of the involved concepts gives rise to difficulties in inferring connections between chemical concepts where no such connections are stated explicitly, something that is possible in formal ontology-backed RDF-based information specifications. Although CML specifications have been increasingly evolving to incorporate elements of the Semantic Web, the lack of widespread adoption of the format, and the limited availability of large-scale CML-based chemical knowledge repositories, have somewhat limited CML-assisted federation of the world of chemical data. Furthermore, the implementation of coverage of additional chemical concepts in most chemical representations requires a formal, rigorous representation specification, complicating the incorporation of data represented using domain-specific representation extensions. We believe that an ideal chemical representation would require no specialized wrapper or interpreter, would be generic such as to allow for facile and conflict-free extensions, would be based on a formal ontology, and would be encoded in a machine-*understandable *(as opposed to simply machine-readable, as in CML) manner and therefore facilitates automated reasoning and data integration.

### Chemical Knowledge Integration

The final point of contention in the world of chemical information archiving is a universal open architecture for chemical databases. Chemical data currently exists in a large collection of application- or institution-specific databases that offer little in the way of an integrative searching approach. In fact, many of these databases expect the end user to rely solely on the information that they provide in their research. In the world where cross-discipline borders are increasingly disappearing, such philosophy should have no place or foothold. It should be possible to seamlessly query for, say, the physical properties of a given chemical entity, as well as for its metabolic fates and toxicity data, and ordering information, all from a single interface. Though a number of databases, such as PubChem [3] and ChemSpider [4] currently offer database cross-links to a number of relevant information providers, the perusal and integration of this information still requires human or human-assisted procedures. Furthermore, until an explicit mapping to a given data source is introduced within a database interface, this data source is inaccessible or difficult to access, making the data practically non-existent to the users of these databases. This situation is complicated by the fact that many data repositories and web services that could potentially generate the required data require unique interfaces and access methods, leaving an immense amount of potentially useful information inaccessible.

With the advent of the Semantic Web, a number of these issues have been addressed. With the concept of linked data and the Resource Description Framework (RDF)-based knowledge representations, a new way of modeling, querying and distributing data became available [15]. With RDF, knowledge is represented in terms of subject-predicate-object triples, where each member of a given triple may be a dereferenceable Universal Resource Identifier (URI) for a particular concept or entity. Thus, what was traditionally referred to as a database, could now be considered a knowledge base, as the initially inert data points were given a machine-understandable meaning through reference to formally specified concepts in supporting ontologies. Thus, two entries from two different knowledge bases could be inferred to relate to the same concept even if no such inference had been explicitly stated, through machine reasoning over axioms in the supporting ontologies. Furthermore, truly integrative queries that could draw on the entirety of the linked data web have now become a reality.

A number of efforts [16-19] have already been successful in the integration of a large portion of chemical information into the linked open data cloud, demonstrating the utility of doing so with successfully fulfilled integrative queries. Such integrative efforts address a number of issues, from the representation of small molecules and their adverse effects to explicit specification of multiple facets of macromolecular structure and interactions. In fact, the chemically-relevant data cloud constitutes a major portion of the entirety of the linked data available on the web [20]. Many of these efforts provide facile means and tools, such as our chemical information RDFization plugin for Open Babel, to represent and distribute any arbitrary chemical information on the Semantic Web [21]. Although these efforts provide a means to of integrating chemical information and of breaking data out of domain or institutional data silos and exposing it for common searches, the triplified data often bears, to a greater or a lesser extent, the same problem as those found in the original databases. For instance, multiple redundant entries for a given molecule, based on database-specific indices may exist without explicit equivalence assertions. Parthood relationships may be missing from such knowledge-bases altogether, and chemical descriptors may be assigned to entities without reference to generating software, experimental conditions, parameters, or data sources. Finally, many of these specifications implement specifications that preclude facile extension of asserted knowledge. For example, a model where an octanol-water partition coefficient is expressed through a predicate and a value, as in '*ethanol hasPartitionCoefficient 1.5*' is not as readily amenable to specification of the conditions under which this value had been generated as its counterpart where the descriptor is given a URI and is fully annotatable with the required information.

### Overview

To rectify the aforementioned chemical information integration problems, we propose CHESS, an RDF-based chemical information specification that is backed by the CHEMINF ontology [22]. Due to the expansive nature of the subject of chemical information representation and the limited space and time to present our work, we shall only focus on selected aspects of semantic chemical information encoding with CHESS, emphasizing principles and consequences rather than specification details. Thus, we shall explore the representation of molecules and all of their constituents with the exception of electrons, representation of chemical descriptors, the consequences of our representation in terms of efficiency of chemical database searches *without specialized cheminformatics plugins*, and finally, we shall briefly cover reaction representation and implications of our representation on reaction candidate selection. By no means do we claim that the representation specification presented here is complete, but would like to rather refer the reader elsewhere for a more detailed and rigorous explanation and implementation examples [23].

## Results and Discussion

### CHESS Representation Overview

The underlying principle in CHESS is to minimize the amount of context-specific rules and regulations, while maximizing the coverage of information represented with the given set of rules. We have also followed an expanded set of principles and requirements in formulating CHESS specification in order to ensure its suitability as a universal chemical exchange language on the Semantic Web, as follows.

1. The most important requirement for CHESS as a universal chemical information framework is the ability to identify and represent chemical entities in a database-, software-, and discipline-independent fashion. For this purpose, we recruit InChI keys and canonical atom numbering arising from the InChI canonicalization algorithm. This also means that atoms, bonds, and functional groups within a molecule have unique and consistent identifiers. Furthermore, all other (physical and informational) entities, such as descriptors, reactions, and macromolecules should also have canonical representations from which consistent identifiers could be obtained.

2. The flexibility and extensibility to support interfacing with and capture of information from a majority of the existing file formats and databases. In previous work, our group has demonstrated OWL serialization of approximately 100 different structure formats with an Open Babel plugin [21]. We continue this trend by providing means to triplify topological and structural information encoded by SMILES, InChI, and SDF chemical formats, as well as by providing an extensible model for specifying chemical properties and descriptors.

3. CHESS should be descriptive enough to provide means for capturing data at various levels of granularity, from atoms to substances, as well as heterogeneity of data from molecular orbitals to chemical reactions. The information thus captured should preserve explicit correspondence to the circumstances of its creation and the other related data points. That is, all the positional descriptors for the atoms in a particular molecule should preserve a correspondence to each other, reconstituting a single conformer, as well as to the parameters and conditions under which they were observed or computed.

4. CHESS should be supportive of Semantic Web Technology-based implementations of basic cheminformatics tasks pertinent to useful analysis of chemical information, such as semantic drug discovery, chemical similarity searching, or reactive pattern matching.

5. Concepts used in CHESS must be backed by a formal ontology in order to facilitate reasoner-mediated integration of chemical information. For the purposes of our specification, we have chosen the CHEMINF ontology.

**Figure 2:**
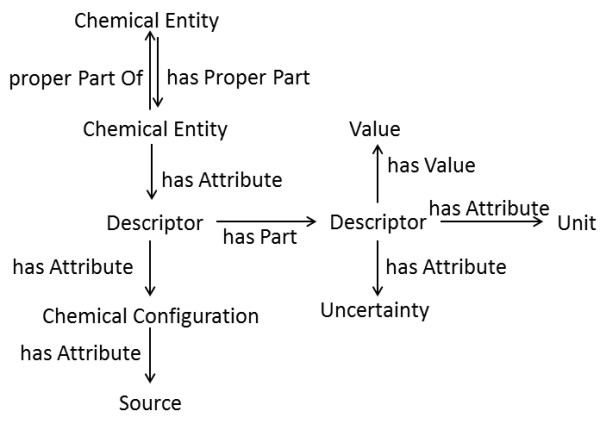
**A simplified overview of the general features of the CHESS specification**. Please note that the chemical configuration is a reflection of the sum of the conditions that may change the value of a given descriptor, as well as the data source.

Thus, the overall specification for CHESS is quite simple, and involves only three broad categories of players: i) chemical entities, comprised of reactions, complexes, molecules, functional groups, bonds, and atoms (but extensible to e.g. electrons, macromolecular assemblies or even subatomic particles), ii) chemical descriptors which could be entity variant or invariant or could be complex and contain multiple CHEMINF ontology-typed descriptors, each with appropriate value, uncertainty, unit, and chemical configuration annotations, and iii) chemical configurations themselves, which reflect upon the sum of relevant conditions under which data has been derived, as well as the sources of data (Figure 2).

Please note that the chemical configuration is a reflection of the sum of the conditions that may change the value of a given descriptor, as well as the data source.

### Molecular Specification

**Figure 3:**
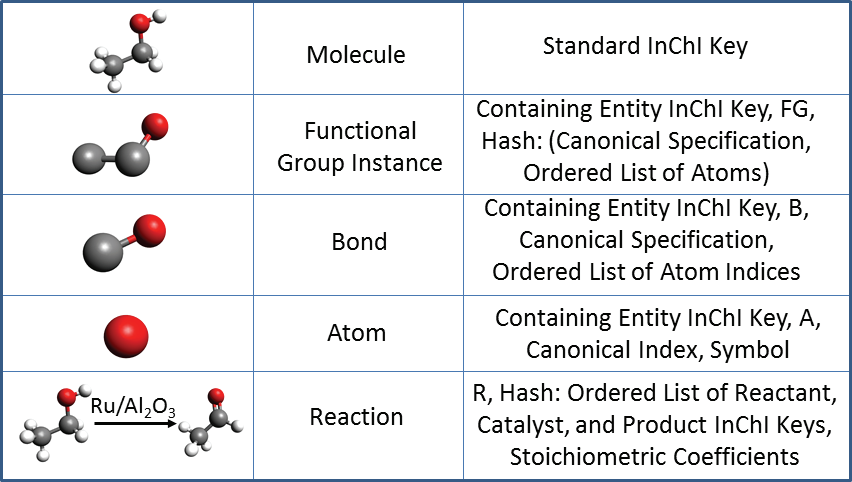
**Principles for generating canonical identifiers for some of the many chemical entity types**. Please note that these identifiers are for instances of chemical entities rather than classes of chemical entities (e.g. all oxygen atoms or all C-O bonds) and necessarily involve the canonical identifier of their containing entity, molecule in this case.

Let us consider in greater detail the methodology of CHESS specification generation on the example of an ethanol molecule. First of all, it is necessary to decide which chemical entities are of interest in a given study. Here, we shall focus on the molecule itself as well as the connectivity of its constituents, including functional groups, bonds, and atoms. In order to respect the first principle of CHESS, it is imperative to generate unique canonical identifiers for each of these entities, according to a set of simple rules that will consistently result in reproducible, database-independent identifiers, some of which we outline here (Figure 3). Please note that although we use our own base URI in our work http://semanticscience.org/resource/CHESS_, we would like to invite the wider chemical community to initiate a discussion on adopting a single, standard base URI for semantic chemistry, to which all entities will be assigned.

In order to enable reasoning and inference over this chemical information, each represented entity also has to be explicitly assigned to a class that is defined within a supporting ontology. This is important to enable querying over broad general concept topics, such as the identification of all instances of oxygen atoms in a given database, or the weakest bond of a particular type in a given molecule, for example. For this purpose, we draw on the concepts present in the Chemical Entities of Biological Interest (CHEBI) ontology and the Semanticscience Integrated Ontology (SIO) [24] to assign general classes to the appropriate chemical entities. For instance, the ethanol molecule may be assigned to a general class of molecular entities (CHEBI:23367), or if the correspondence is present, to a more specific class of molecules, such as that of primary alcohols (CHEBI:15734). Functional groups or molecular substructures may also be assigned to a general class within the CHEBI ontology (e.g. CHEBI:33249), bonds to an appropriate SIO class (SIO_011118), atoms to their appropriate types in CHEBI (e.g. CHEBI:25805 for oxygen), and reactions to the *chemical reaction *class in SIO (SIO_010345). The end-user is not limited to the pre-defined classes in the SIO or CHEBI ontologies. Because these classes are fully extensible, it is possible to define a more specific class for each of the chemical entities presented here. For instance, one may extend SIO's *covalent chemical bond *(SIO_011118) to create a subclass corresponding to carbon-oxygen single bonds, or extend the broad class of functional groups or molecular substructures in CHEBI to correspond to a class of substructures that satisfy or exactly match a general pattern, such as CCO, as we shall see later.

Though functional group or substructure specification is optional, as is that of any component not relevant to the chemical information represented, in order to demonstrate chemical database searching and reaction candidate matching in this study, we have automatically generated a set of unique atom-centric molecular sub-graphs consisting of heavy atoms and containing first, second, and/or third neighbours of each heavy atom in a given molecule. For example, the oxygen-centered fragmentation products of *n*-propanol of connectivity 1, 2, and 3 are the hydrogen-suppressed graphs CO, CCO, and CCCO, respectively. These fragments are given unique and reproducible identifiers, based on fragment chemical graph structure and the canonical indices of the member heavy atoms, as well as their molecule of origin. It must be noted that the CHESS specification itself is not limited to this automatically generated set of fragments, but rather we are using this set it in order to achieve further goals of enabling chemical similarity searching. Customized fragment or functional group annotations, just like annotations of any other type, may be added to the triple store containing the chemical entities under investigation at any time.

To complete the semantic description of the molecular skeleton, mereological relationships between the various sub-components of a given entity have to be asserted. These relationships are captured with *has proper part *(SIO_000053). Based on this information, the complete chemical graph can be reconstituted, and our chemical entity under investigation is ready for further annotation or querying. Here, we provide examples of the specification for each entity discussed (Appendix 1).

Appendix 1. RDF/N3 CHESS representation of ethanol and its constituent parts.

@prefix rdf: <http://www.w3.org/1999/02/22-rdf-syntax-ns#>

@prefix sio: <http://semanticscience.org/resource/SIO_>

@prefix chess: <http://semanticscience.org/resource/CHESS_>

@prefix chebi: <http://purl.org/obo/owl/CHEBI#>

#Specify ethanol as a CHEBI molecule using its InChI key

chess:LFQSCWFLJHTTHZ-UHFFFAOYSA-N

rdf:type chebi:CHEBI_23367.

#Specify ethanol's alcohol group as a CHEBI organic group instance.

chess:LFQSCWFLJHTTHZ-UHFFFAOYSA-N-FGd787664f213a8ffded68d4d945d012b0cfaf7aa4

rdf:type chebi:CHEBI_33247.

#Specify ethanol's C-O bond as an instance of SIO's single bond.

chess:LFQSCWFLJHTTHZ-UHFFFAOYSA-N-BCO23

rdf:type sio:010498.

#Specify ethanol's oxygen atom as an instance of CHEBI's oxygen atom.

chess:LFQSCWFLJHTTHZ-UHFFFAOYSA-N-AO3

rdf:type chebi:CHEBI_25805.

#Assert proper part between the oxygen atom and the molecule.

chess:LFQSCWFLJHTTHZ-UHFFFAOYSA-N

sio:000053 chess:LFQSCWFLJHTTHZ-UHFFFAOYSA-N-AO3.

### Semantic Web-Enabled Cheminformatics: Chemical Searching

Since the specification we have so far described can be used to reconstitute the molecular graph, we can now venture to study the most optimal approaches to enabling some of the most common tasks in cheminformatics. Here, we shall initially focus on the classical task of chemical database searching by chemical similarity. Although it is possible to invoke plugins or intermediary specialized software to enable rapid database searching, we argue that unlike many other information representations and formats, CHESS allows us to fully represent the chemical graph and should therefore be readily amenable to graph manipulation and similarity searching. Furthermore, we believe that, within the limit of providing an expressive enough specification of chemical entities under investigation, the efficiency of querying a knowledgebase created using a given knowledge representation is a good indicator of the efficiency of the representation itself.

Chemical similarity searching is a complex topic that lies at the heart of cheminformatics and can be carried out in a wide variety of ways to address a number of problems. Because our specification provides us a complete chemical graph description, we have chosen to first attempt a Semantic Web-native solution for the substructure matching problem, using description logic-safe rules [25] and SPARQL query language-based queries [26] on the molecular structure. As a benchmark, we have used representative subsections of the LIPIDMAPS database [27] of lipids and their structures of sizes 10, 100, and 10000 molecules in partitions DB10, DB100, and DB10000, respectively. As query graphs, we have used a series of linear carbon chains, from ethyl to pentyl, cyclopentene, and a number of lipid-related functional groups, including glycerol, sterol, a fatty acyl moiety, a sphingolipid moiety, and a prenol lipid moiety.

While carrying out our tests, we have become aware of the complexity of modeling bonds as explicit entities. While this specification allowed for facile annotation of bonds with properties and descriptors, it resulted in significant search performance hits, forcing us to reconsider elements of our specification. As a result, we have created a test set where, apart from specifying explicit bonds, we also linked bonded atom instances with the appropriate bidirectional relationships that corresponded to bond type (single, double, triple, or aromatic). This improved performance considerably and allowed us to carry out our tests. Herein we find another demonstration of the versatility of semantically enabled information representations: we have been able to extend and amend the information in our knowledge repository without much additional effort or adverse effects on the knowledge repository. So long as our specification is consistent with the formal axioms underpinning the concepts in a supporting ontology, there is no barrier preventing us from extending our specification indefinitely.

One may search for molecules containing a particular sub-graph using the explicit specification of the structure of the sought molecular sub-graph as a SPARQL query (Appendix 2). It must be noted that answering this query in a typical SPARQL engine involves the exhaustive examination of all the candidate molecules that may potentially contain a collection of atoms that satisfy the laid out bonding criteria. Unfortunately, since the SPARQL query engines currently available have not been explicitly optimized chemical searching needs, they often lack many of the mechanisms developed over the past decades to accelerate the solution of this problem, and resemble the brute force approach to graph matching more closely.

Appendix 2. An automatically generated (based on graphical user input) SPARQL query to identify all molecules containing an ethyl subgraph, altered for improved readability. Note the use of 'has single bond with' direct atom relationship to improve query performance.

prefix rdf: <http://www.w3.org/1999/02/22-rdf-syntax-ns#>

prefix sio: <http://semanticscience.org/resource/SIO_>

prefix chess: <http://semanticscience.org/resource/CHESS_>

prefix chebi: <http://purl.org/obo/owl/CHEBI#>

SELECT distinct ?m

WHERE {

?m rdf:type chebi:25367. #molecule

?a0 rdf:type chebi:27594. #carbon atom

?a1 rdf:type chebi:27594. #carbon atom

?m sio:0000053 ?a0. #has proper part

?m sio:0000053 ?a1. #has proper part

?a0 chess:'has single bond with' ?a1.

FILTER(?a0 != ?a1).

}

Description logic-safe rules may be used to reason about instances of an OWL-DL ontology where the description follows a graph-like pattern instead of the general 'tree-like' expression. In this way, one can combine classical DL-reasoning with graph-like descriptions to classify molecules as more specific kinds of compounds, provided that they satisfy certain sub-graph conditions. For instance, an ethyl compound would be a kind of a molecule that contains an ethyl group with two carbon atoms linked together by a single bond. Overlooking for the sake of simplicity of the immediate example the other necessary conditions, such as the lack of branching or membership in a ring, this may be represented as the following rule (Appendix 3).

Appendix 3. An automatically generated (based on graphical user input) dl-safe rule for identifying an ethyl group-containing molecule, altered for improved readability.

'molecule'(?m1), 'carbon atom'(?a0), 'carbon atom'(?a1),

'is part of'(?a0, ?m1), 'is part of'(?a1, ?m1),

'single bond'(?a0, ?a1), 'different from'(?a0, ?a1)

-> 'ethyl containing molecule'(?m1)

**Figure 4:**
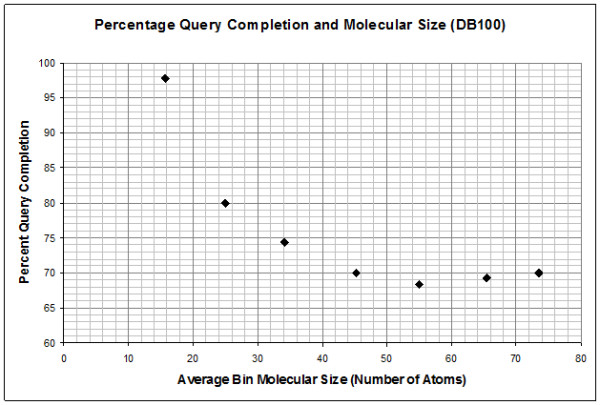
**SPARQL query completion and average molecular size in DB100**.

Both, the SPARQL querying, and the rule-based reasoning, completed in the allocated time of 120 seconds on the simple carbon chain-based queries. However, the iterative identification of compounds with more complex substructures, such as those relevant in the classification of lipids, fails with rule reasoning using DB10 for the majority of molecules in the database. In all cases, we observed either memory exhaustion (despite having allocated 5GB of memory), or premature termination. Note that the time limit was imposed after observing that even if Pellet (see chapter "Query Testing") was allowed to reason over an unconstrained amount of time, this would only delay termination due to memory exhaustion. SPARQL-based query answering, on the other hand, succeeds in identifying molecules containing many fatty acyl patterns, but fails between 20-40% of the time in DB10, and between 60-70% of the time in DB100 for prenol lipid, sphingolipid and sterol lipid SPARQL queries. Generally, SPARQL query completion exhibits a dramatic drop across all test cases with increasing number of atoms in the database molecules searched at a given time (Figure 4). In addition to this discouraging result, the increase of search pattern size or complexity had an even more profound effect on query completion, from exceeding the completion time limit to SPARQL query engine-triggered query termination due to the query computational load exceeding any reasonable expectations, of 11 years, for example (results not shown).

**Figure 5:**
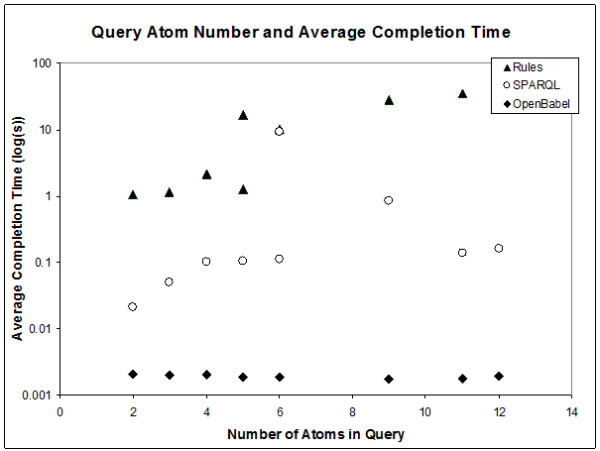
**General performance trends for the two query modes relative to Open Babel matching**. Note that the two points at six query atoms for SPARQL queries is due to alternate structures: cyclical structures are more time-consuming than linear ones.

In terms of performance for queries that were successful, both DL-safe rule reasoning and SPARQL-based querying are 1-4 orders of magnitude slower than using Open Babel, and DL-safe rules are 1-2 orders of magnitude slower than SPARQL queries. By comparing the number of atoms in the query structure to the completion time we observe that Babel performance is linear, but the performance of searching with both, SPARQL and DL-safe rules, appears exponential or parabolic (Figure 5).

Given these discouraging results, even for an extremely small data set, it was clear that an alternative approach or a redefinition of the problem to a more manageable one was in order. As we have discussed, by incorporating functional group/sub-structure information at the time of creating the molecular specification, or by adding it to the existing representation stores, we can redefine our searching problem. By doing so, it becomes possible to use the same device as the one used in fingerprint-based searches: in order to identify database molecules that are similar to the query, it is simply necessary to rank them in terms of the number of substructures that belong to the same class as those in the query. The resultant query exhibits relatively rapid completion times that depend weakly on the complexity of the queried structure and linearly on database size (Appendix 4). Overall, the completion time of this query for DB10000 is well within the stipulated time limit for all the queries attempted (results not shown). Although this is encouraging, many commercially and scientifically important chemical databases enumerate several orders more molecular entities, casting a shadow over the applicability of this approach to large-scale applications, until a more detailed study of search performance demonstrates otherwise.

Appendix 4. The general form of the SPARQL query to identify molecules similar to a queried molecule, which can be obtained from graphical user input. Query amended for readability.

PREFIX sio: <http://semanticscience.org/resource/SIO_>

PREFIX chess: <http://semanticscience.org/resource/CHESS_>

select distinct ?x count(?y) where {

?x rdf:type chebi:25367. #molecule

?x sio:0000053 ?y. #has proper part

FILTER (?y = chess:FG1 || ?y = chess:FG2)

}

ORDER BY (100/(count(?y))) LIMIT 100

Although we have not tested the more efficient tools currently available, and although the performance of SPARQL query engines [28] and machine reasoners [29] continue to improve, we can see that chemical similarity searches that rely solely on existing Semantic Web tools are possible, but may be problematic for very large chemical knowledge bases. Certainly, we believe that optimization of Semantic Web-based chemical searching solutions with the latest and the most efficient tools, as well as the application of existing tools to very large (over a million molecules) stores of chemical information, warrants further elaboration in an expanded, separate study. Other, significantly more rapid searching solutions that draw on both, the Semantic Web technologies, and existing methodologies, are also available. For example, it is possible to create custom SPARQL functions that encapsulate specialized code to either carry out pairwise similarity comparisons between a query and database molecules by reconstructing and comparing chemical graphs from InChI, SMILES, or SMARTS annotations. It is also possible to envision specialized functions to carry out simple Tanimoto comparison of query and database molecule fingerprint strings. Finally, semantically-enabled web services may be used to carry out such searches [30]. However, the purpose of this excursion has not been the immediate creation of a solution that could outperform specialized and streamlined code carrying out optimized sub-graph detection or similarity calculations on in-memory stores of molecular fingerprint strings [31]. Rather, the fact that it is possible to attempt parser- and specialized tool-free analysis and integration of chemical data, demonstrates the potential, power, and versatility of investigations afforded by adopting a semantic specification of chemical entities.

### Chemical Descriptor Specification

Having explored in detail semantic specification of various chemical entities and their parts, let us turn our attention to their annotation. Chemical annotations may be classified into two broad types: those that are dependent solely on the composition and the nature of a given entity (e.g. standard InChI strings or heavy atom count), and those that capture empirically or theoretically derived data that varies depending on the circumstances of the chemical entity or data observation (e.g. solubility, computed logP). In CHESS, the two cases are specified through a common approach stipulated in the CHEMINF ontology with one major difference: non-constant descriptors are assigned to a *chemical configuration *that reflects upon the circumstances of descriptor creation and the circumstances of the entity to which these descriptors refer. For instance, the calculated free energy of formation of a molecule in gas phase depends not only upon the computational package employed to derive the value and parameters like temperature or the level of theory used, but also on the geometric configuration of the molecule.

Unlike chemical entities, it is not absolutely crucial that descriptors have canonical and reproducible names, as they are rarely used as focal points around which other annotations are integrated. That is, although it is important to ensure that descriptor identifiers do not clash, thus overwriting or contradicting any data previously assigned to a given descriptor, the precise form of descriptor identifier and its canonical nature are of secondary importance. In this work, invariant descriptors receive unique and canonical identifiers, based only on a hash of essential descriptor components: value, uncertainty (if any), and units. Variable descriptors, on the other hand, derive their identifiers from these parameters in addition to the canonical identifier of their corresponding chemical configuration, itself derived by hashing values of its annotations, presented in lexicographical order. Multi-component descriptors that include other descriptors, such as the non-invariant positional descriptor for atoms, receive an identifier that is a hash of the canonical identifiers of constituent descriptors in lexicographical order instead of a direct hash on the value, uncertainty, and unit annotations. In both cases, the resultant hash is appended to the identifier of the entity to which these descriptors refer in order to obtain the final identifier.

**Figure 6:**
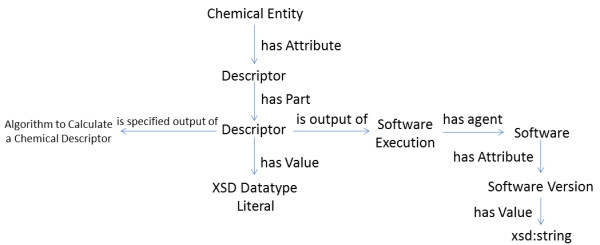
**A simplified descriptor specification, as per the CHEMINF ontology approach**.

The scheme for specifying descriptors is quite simple (Figure 6). Descriptors may contain other descriptors and may have an unlimited number of annotations, such as units and uncertainty, but must have a value assigned. Furthermore, CHESS follows the CHEMINF approach to modeling computationally-derived descriptor provenance. Thus, a chemical descriptor is an information content entity that is a specified output of an algorithm (e.g. Mannhold logP algorithm [32]), and an output of a parameterized or non-parameterized software execution process. This process has to be annotated with the identity of the software agent employed, which may be further annotated with e.g. software version, and any parameters (formally defined in an ontology) used in carrying out the calculation. Experimentally-derived descriptors follow a similar scheme, but refer to experimental observations and processes.

In addition to this specification, descriptors are also annotated with a chemical configuration. This concept is useful not only as a nodal point for storing source and other provenance information, but also in uniting the descriptors that have been derived under a uniform set of conditions and for the system under investigation that is in a given, well-defined state. Because chemical configurations are also, in part, specific to a geometric configuration of a given entity, it also allows a non-confounded aggregation of data, e.g. in representing atomic coordinates for multiple conformations of the same molecular entity or investigating the thermodynamic properties of molecules that may have different electronic configurations (e.g. singlet or triplet oxygen) or molecules that may be exposed to different temperatures. This approach also does away with data retrieval and integration complexities arising from heterogeneously derived information attached to a single chemical entity in certain databases, manifested in the requirement for extensive involvement of a human expert to identify a set of descriptors suitable for a particular comparison. So long as chemical information is properly annotated, descriptors specified with our approach (Appendix 5) are readily amenable to facile querying and retrieval (Appendix 6), significantly reducing the workload on an individual researcher. Alternatively, querying the knowledgebase for all descriptors from different databases that refer to the same geometric configuration or experimental conditions, is also possible.

Appendix 5. Sample specification of variable descriptors with reference to a chemical configuration, amended for readability.

@prefix rdf: <http://www.w3.org/1999/02/22-rdf-syntax-ns#>

@prefix sio: <http://semanticscience.org/resource/SIO_>

@prefix chess: <http://semanticscience.org/resource/CHESS_>

@prefix chebi: <http://purl.org/obo/owl/CHEBI#>

#Specify an oxygen atom in ethanol.

chess:LFQSCWFLJHTTHZ-UHFFFAOYSA-N rdf:type chebi:CHEBI_23367.

chess:LFQSCWFLJHTTHZ-UHFFFAOYSA-N-A3O rdf:type chebi:CHEBI_25805.

chess:LFQSCWFLJHTTHZ-UHFFFAOYSA-N sio:SIO_000053 :LFQSCWFLJHTTHZ-UHFFFAOYSA-N-AO3.

#Three-dimensional coordinate specification.

:DCCC rdf:type sio:3D Cartesian coordinate.

:LFQSCWFLJHTTHZ-UHFFFAOYSA-N-A3O sio:'has attribute' :DCCC.

:DXXX rdf:type sio: 'x Cartesian coordinate'.

:DCCC sio:'has direct part' :DXXX.

:DYYY rdf:type sio: 'y Cartesian coordinate'.

:DCCC sio:'has direct part' :DYYY.

:DZZZ rdf:type sio: 'z Cartesian coordinate'.

:DCCC sio:'has direct part' :DZZZ.

#Define the x Cartesian coordinate.

:DXXX sio:'has value' "1.55".

:DXXX sio:'has unit' <http://purl.org/obo/owl/UO#UO_0000019>.

#Define a chemical configuration.

:CCXXX rdf:type sio:000659.

:CCXXX sio:'has provider' "http://pubchem.ncbi.nlm.nih.gov."

#Link the chemical configuration to the 3D Cartesian coordinate.

:DCCC sio:has attribute :CCXXX.

Appendix 6. Sample query of variable descriptors that would retrieve coordinate information for all atoms of ethanol that originate from PubChem, amended for readability.

prefix rdf: <http://www.w3.org/1999/02/22-rdf-syntax-ns#>

prefix sio: <http://semanticscience.org/resource/SIO_>

prefix : <http://semanticscience.org/resource/CHESS_>

prefix chebi: <http://purl.org/obo/owl/CHEBI#>

select ?a, ?x,?y,?z where {

?d rdf:type sio:'3D Cartesian coordinate'.

?d sio:'has provider' "http://pubchem.ncbi.nlm.nih.gov".

?a rdf:type chebi:atom.

?a sio:'has attribute' ?d.

:LFQSCWFLJHTTHZ-UHFFFAOYSA-N sio:'has proper part' ?a.

?d sio:'has direct part' ?xx.

?xx sio:'has value' ?x.

?d sio:'has direct part' ?yy.

?yy sio:'has value' ?y.

?d sio:'has direct part' ?zz.

?zz sio:'has value' ?z.

}

### Chemical Information Integration

Having described at length the representation of chemical information and various entities, let us consider the practical effects of decisions taken in CHESS on information integration. Although we have generated and successfully integrated moderately-sized subsections of various publically accessible chemical databases, the overall effect of our specification can be illustrated on the example of a limited set of molecules present in multiple databases or having descriptors created by different computational procedures. To demonstrate the facile cross-database and cross-study information integration afforded by consistent canonical entity identifiers, consider two instances of the same compound, antidepressant melitracene, in two different databases, PubChem and ChEMBL. Although these entries are cross-linked in their respective databases, the comparison and integration of information regarding this entity involves a procedure that requires a degree of human involvement, especially if this entry has to be further cross-linked to another entry in any number of other databases, each having unique data fields or approaches to data presentation and representation.

With CHESS, it is possible to independently encode the information in each repository in separate RDF graphs. However, because chemical entity URIs for melitracene are the same in all cases http://semanticscience.org/resource/CHESS_GWWLWDURRGNSRS-UHFFFAOYSA-N, all of these graphs effectively collapse into a single graph, with a SPARQL query to retrieve information relevant to melitracene capable of seamlessly drawing on the entirety of the chemical knowledge, without regards to originating database- or software-specific integration barriers. At the same time, this allows us to address issues relating to data correspondence, as bonds with the same URI are assured to be the same entity. This eliminates the need for substructure matching or other intermediate steps in carrying out cross-database comparisons. This ability may be especially useful in cases where, for example, multiple computational experiments are performed and the computed bond lengths need to be compared to the experimentally observed bond lengths or to the results of other computational experiments.

As a simple demonstration of this capability, we have generated a small set of 90 CHESS-encoded antidepressants containing selected information from two databases, as well as three different computational packages. The descriptors we have represented in this knowledgebase were of relevance to satisfying Lipinski's Rule of Five [33], allowing us to potentially pool all of the available information in these disparate databases and computational experiments to address the question of whether the compounds in our knowledgebase were, in fact, drug-like. For each source of information, a number of descriptors were intentionally left out to demonstrate the assurance of information complementarity and preservation of information correspondence with the CHESS representation. To test this, we have created a custom class with a formal definition corresponding to Lipinski's Rule of Five and reasoned over it using the Pellet reasoner plugin (version 1.4) [34] in Protégé software [35] (version 4.0, build 115). As this class was correctly populated with 88 instances of chemical entities satisfying the Rule of Five, it became apparent that consistent molecular identifiers permitted the effective collapse of the multiple knowledge sources to provide the information necessary in order to fulfil the classification and identify drug-like chemical entities. Furthermore, the correspondence of the molecular descriptors specified in our knowledgebase was adequately preserved.

Appendix 7. The formal axiomatic definition of a class of chemical entities that satisfy Lipinski's Rule of Five, using CHEMINF concepts (specified using the Manchester OWL syntax).

'chemical entity' and 'has attribute'

some ('mass descriptor' that 'has value' some double[<500.0])

and 'has attribute'

some ('hydrogen bond acceptor count' that 'has value' some int[<=10])

and 'has attribute'

some ('hydrogen bond donor count' that 'has value' some int[<=5])

and 'has attribute'

some ('logP descriptor' that 'has value' some double[>-5.0,<5.0])

### Variable Level Granularity Semantically Enriched Annotations and Queries

We have established that CHESS addresses the problem of seamless data integration across multiple sources of information by demonstrating a query that reproduces a common Rule of Five filter, on an integrated data set from three different sources. However, this kind of filtering can currently be readily carried out for most chemical databases through their search engine interfaces provided by the suppliers of such databases, such as PubChem. In the absence of such exposed search interfaces, however (e.g. when there is no option to restrict search results based on molecular mass), users of these chemical information repositories are faced with the task of manually parsing or calculating the chemical information that is needed for answering their research question. In addition to this, annotations of existing entities with new information in smaller studies are often lost due to the database barriers discussed at length in this work, or the practicality of publishing 'smaller' scientific data as an accessible database that is open to querying. Finally, the level of annotation granularity allowed in a given database may be insufficient for a particular application. That is, while data on individual atoms and bonds certainly exists in PubChem, it is impossible to refer to, retrieve, or annotate these individual entities, at least not in a manner that would immediately meaningfully connect the annotations generated as a part of a given study with a given PubChem entity and would allow these annotations to be discovered and queried.

In contrast, CHESS is flexible and extensible: annotations and information represented in CHESS is assured to be searchable, no matter what information the database vendor feels like exposing. So long as there is chemical information represented in CHESS, it is subject to logical queries and semantic integration. Furthermore, all chemical entities, such as atoms, bonds, and molecules, can be fully annotated using a range of vocabularies, and these annotations can be linked directly to the entities being annotated, even if the original entity and its annotations reside in different RDF graphs. With CHESS, it is now possible to facilitate open publishing of scientific information and assure that the precious scientific knowledge is preserved, no matter how small a study has been carried out.

To demonstrate the benefits of this approach, let us consider a practical case that is impossible to address with currently existing chemical databases. For this, let us examine phenolic antioxidants, which constitute an important class of molecules that are used in industrial processes and as nutritional supplements to help alleviate the damaging effects of free radicals, such as lipid peroxidation in oils. It has been shown that Bond Dissociation Enthalpies (BDEs) of the phenolic O-H bonds can be used as excellent predictors of the potency of a phenolic compound. When designing phenolic antioxidants for biological systems (e.g. humans), one has to be mindful that the BDE of the phenolic O-H bond has to be higher than that of the weakest O-H bond in ascorbate (67 kcal/mol) to allow for biological antioxidant recycling, but lower than that of the O-H bond of α-tocopherol (78 kcal/mol) to surpass the potency of existing physiological antioxidant defences [36]. While the accurate annotation and searching of bond-level information is currently impossible in major chemical information repositories, we have developed a demonstrative set of chemical entities with O-H BDE information annotation (available from our companion website [23]), including the computational method, software, and some of the parameters used to compute this information for ethanol (Appendix 8).

Appendix 8. A representative portion of the CHESS specification of a ethanol, its constituent OH bond, and the parameters used in BDE calculation for this bond.

@prefix rdf: <http://www.w3.org/1999/02/22-rdf-syntax-ns#>

@prefix sio: <http://semanticscience.org/resource/SIO_>

@prefix : <http://semanticscience.org/resource/CHESS_>

@prefix chebi: <http://purl.org/obo/owl/CHEBI#>

@prefix cheminf: <http://semanticscience.org/resource/CHEMINF_>

#Specify an OH bond in ethanol.

:LFQSCWFLJHTTHZ-UHFFFAOYSA-N rdf:type chebi:CHEBI_23367.

:LFQSCWFLJHTTHZ-UHFFFAOYSA-N-BOH7 rdf:type sio:010498.

:LFQSCWFLJHTTHZ-UHFFFAOYSA-N sio:SIO_000053 :LFQSCWFLJHTTHZ-UHFFFAOYSA-N-BOH7.

#BDE Annotation Specification.

:DBDE rdf:type cheminf:000252. #BDE Descriptor.

:LFQSCWFLJHTTHZ-UHFFFAOYSA-N-B)H7 sio:'has attribute' :DBDE.

:DBDE sio:'has value' "104.5".

:DBDE sio:'has unit' "kcal/mol".

#BDE Provenance Specification.

:DBDE cheminf:'is specified output of' :AM1.

:AM1 rdf:type cheminf:000144.

:DBDE cheminf:'is output of' :ExecutionXYZ.

#Parameterized software execution.

:ExecutionXYZ rdf:type cheminf:000147.

:ExecutionXYZ sio:'has attribute' :TempX #Temperature parameter.

:TempX sio:'has value' "298".

:TempX sio:'has unit' <"http://purl.org/obo/owl/UO#UO_0000012">

#Software Package Used

:ExecutionXYZ sio:'has participant' :MOPACV7.

:MOPACV7 rdf:type sio:'software application'.

:MOPACV7 sio:'has attribute' :VMOPAC7.

:VMOPAC7 rdf:type sio:'software version'.

:VMOPAC7 sio: 'has value' "7.1.11".

Please note that this representation was simplified and modified to improve readability.

A knowledgebase with such information could have been published and shared as an outcome of any of a number of studies on this subject, with annotations attached directly to existing chemical entities in one of the major chemical databases. Instead, this information was sealed away in a series of PDF and HTML documents, accessible only to those with the time and resources to locate and read them. In order to retrieve *phenolic *antioxidants with potential applications in biological systems, one could combine the molecular structural features (the presence of a phenol group) with thermochemical annotation information on the OH bond. Furthermore, since computationally derived thermochemical parameters are best compared when the method, software, and parameters used to derive them are uniform, we may include these requirements in our query to retrieve uniform and useable information. Such an integrative query will provide all the potentially potent novel phenolic antioxidants from a chemical knowledgebase (Appendix 9). Please note that such integrative, variable-granularity, and flexible queries are impossible for the major conventional chemical information repositories. Truly, the imagination of CHESS users is the limit for the expressivity and the semantic enrichment of the represented chemical information.

Appendix 9. Sample query that may be carried out across a single or multiple SPARQL endpoints to retrieve all potentially promising phenolic antioxidants from an annotated compound collection, amended for readability.

prefix rdf: <http://www.w3.org/1999/02/22-rdf-syntax-ns#>

prefix sio: <http://semanticscience.org/resource/SIO_>

prefix : <http://semanticscience.org/resource/CHESS_>

prefix chebi: <http://purl.org/obo/owl/CHEBI#>

select ?m, ?value where {

?m rdf:type chebi:CHEBI_23367. #A molecule

?m sio:'has proper part' ?f . #that has a moiety

?f rdf:type :FGPhenol #which is phenol.

?m sio:'has proper part' ?b . #This molecule

?b rdf:type sio:010498. #also has a single bond

?b sio:'has proper part' ?o. #which has atoms O and H.

?o rdf:type chebi:25805.

?b sio:'has proper part' ?h.

?h rdf:type chebi: 49637.

?b sio:'has attribute' ?d. #This bond has

?d rdf:type cheminf:000252. #a BDE descriptor

?d cheminf:'is specified output of' :AM1. #computed with AM1

?d sio:'has unit' "kcal/mol". #in units of kcal/mol

?d sio:'has value' ?value . #and with a value

FILTER(?value > 67 && ?value < 78) #between 78 and 67.

}

### Representing and Querying Reactive Transformations

As a final demonstration of usability of CHESS-encoded chemical information, let us consider reaction representation and querying. As mentioned earlier, reactions in CHESS are considered to be chemical entities, whose identifiers come from their participants and stoichiometry. Though reactions may or may not take place under a certain range of circumstances, we model them as abstract processes that exist without regards to their likelihood of occurring. Because as long as a specified reaction respects universal principles, such as the conservation of matter and energy any reaction may happen at any time (some are more likely than others), we maintain that the sole features relevant to unique reaction specification are the chemical identities and stoichiometries of the participating chemical entities (Figure 3).

**Figure 7:**
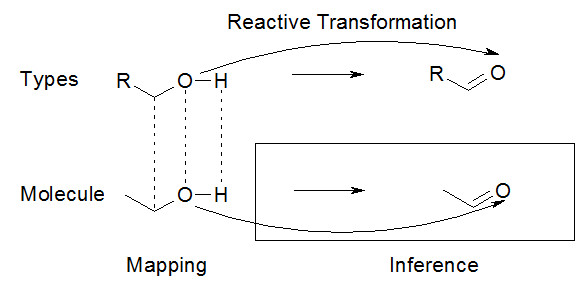
**Mapping components of an instantiated molecule to reactive transformation definition participants can enable a range of inferences relevant to predicting reaction outcome**.

In general terms, there are two types of reactive transformations specified: those involving generic chemical moieties and their transformations and those that involve precisely defined chemical entities. Both cases may be represented in CHESS in a uniform and consistent fashion. The only difference between the two cases is the specification of the entities involved, which in the former case are of the functional group/substructure type, and in the latter are instantiated molecular entities. In either case, the most important feature that decides whether a molecule will undergo reactive conversion is the presence of a characteristic functional group within the substrate molecule. That is, reactions can be viewed as transformations between the various isolated regions, or functional groups, with the rest of the molecule attenuating or increasing reactivity. The presence of the requisite functional group through explicit mapping of constituent atoms and bonds makes it possible to infer the role of a given molecule and its components in a reactive process (Figure 7).

The amount of information regarding a reactive transformation that could be inferred is entirely dependent upon the amount of information present in the reference knowledgebase and in the reaction specification RDF graph. For a demonstrative example, let us consider a transformation of primary alcohols to aldehydes, which could be catalyzed by an inorganic catalyst or by an enzyme. Since generic functional group transformations are involved, reaction specification would involve functional group types and atom types, rather than corresponding instances. Substrates are linked to the reaction using *has input *(SIO_000230), products using *has product *(SIO_000312) and catalysts using *has agent *(SIO_000139). Further, to maintain a record of the correspondence of every transforming entity, the transformations would be specified with *transforms into *(SIO_000655), which can operate upon whole molecules, functional groups, and atoms (Appendix 10).

Appendix 10. Sample reaction definition of CCO functional group transformed into CC = O functional group with CHESS in N3-turtle, amended for readability.

@prefix : <http://semanticscience.org/resource/CHESS_>

@prefix sio: <http://semanticscience.org/resource/SIO_>

@prefix chebi: <http://purl.org/obo/owl/CHEBI#>

#Reaction inputs, outputs, and agents.

:RXXX rdf:type sio:'chemical reaction'.

:RXXX sio:'has input' :FGXXX.

:RXXX sio:'has input' :FGXXXA3O.

:RXXX sio:'has product' :FGYYY.

:RXXX sio:'has product' :FGYYYA3O.

:RXXX sio:'has agent' :KJFCCLURYALNSL-UHFFFAOYSA-N. #Ru/Al_2_O_3_

#Transformations of functional groups.

:FGXXX rdf:type chebi:'organic group'.

:FGYYY rdf:type chebi:'organic group'.

:FGXXX sio:'transforms into' :FGYYY.

#Transformations of atom types in these groups.

:FGXXXA3O rdf:type chebi:'oxygen atom'.

:FGXXX sio:'has proper part' :FGXXXA3O.

:FGYYYA3O rdf:type chebi:'oxygen atom'.

:FGYYY sio:'has proper part' :FGYYYA3O.

:FGXXXA3O sio:'transforms into' :FGYYYA3O.

**Figure 8:**
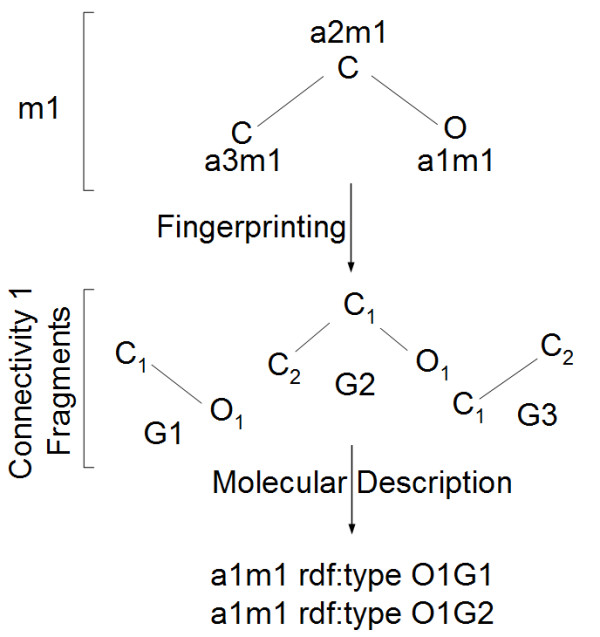
**A modification of molecular fingerprinting used for the complete description of chemical structures in terms of an exhaustive list of functional groups**. Molecular fragments resulting from fingerprinting (G1, G2, G3) may be stored and treated as descriptive functional groups, along with user-submitted ones.

To enable reaction matching, it is necessary to also obtain the atom and functional group typing information for the chemical entities in the CHESS knowledgebase. For this purpose, one may use an extension of the described atom-centric fingerprinting procedure by typing atom and functional group instances to the generic classes of atoms and functional groups they instantiate. Thus, instead of merely asserting membership of group LFQSCWFLJHTTHZ-UHFFFAOYSA-NFGXYZ in the ethanol molecule (where to save space, XYZ is the appropriate hash), it would be typed to the FGXXX functional group. A similar procedure has to be carried out for atoms if atom-centric searches need to be enabled, e.g. for tracing the precise passing of atoms between molecules, and checking the metabolic history of every single atom (Figure 8). As mentioned earlier, this process may be carried on at any point, and the knowledgebase may be amended with the required information.

The resultant specification of reactions and chemical entities enables simple, yet powerful SPARQL-based queries, e.g. to find the potential reactions of a given molecule and identify the atoms and functional group instances involved in these reactions (Appendix 11).

Appendix 11. A query that will return all the reactions that ethanol may potentially be involved in, amended for readability.

prefix : <http://semanticscience.org/resource/CHESS_>

prefix sio: <http://semanticscience.org/ontology/sio.owl#>

prefix chebi: <http://purl.org/obo/owl/CHEBI#>

select * where {

?reaction rdf:type sio:'chemical reaction'.

?reaction sio:'has input' ?FGAtom.

?FGAtom rdf:type chebi:'atom'.

?MAtom rdf:type ?FGAtom.

?MAtom sio:'is part of' :LFQSCWFLJHTTHZ-UHFFFAOYSA-N

}

### Supporting Tools and Interfaces

An evolving project site with supporting information, graphical user interface-backed tools, sample RDF specifications and data sets (including a sample set of ~17000 ChEBI compounds), as well as further information, is available [23]. The implementation of CHESS provided on the website has a scope limited to compounds that can be well-represented with current version of InChI. Therefore, we currently exclude polymers with repeating units of arbitrary length and Markush structures from the scope of CHESS, for example. On the other hand, organometallic compounds and metals that can be represented with InChI are included within our scope. While there is no theoretical limit to the size of a compound that can in principle be represented using CHESS, our sample implementation is practically limited (by the available computational resources on the server) to lower molecular weight compounds.

## Conclusions

Unfortunately, many of the large chemical databases currently do not possess the means of chemical data integration and federation. Either for historical reasons or for efficiency improvement, a large number of these databases have been purpose-built for capturing data within a particular domain, and without much consideration of trans-domain knowledge aggregation. This further complicates the task of database integration and poses as an obstacle to productive chemical research. Fortunately however, the Semantic Web provides an excellent opportunity for significantly simplifying this problem with the appropriate data representation and sufficiently advanced data conversion tools.

In this work, our principal goal has been to present a novel chemical representation formalism that draws on the Semantic Web principles. We have attempted to make a compelling case for a universal semantic specification of chemical entities in cheminformatics by demonstrating the power, integrative capacity, and the flexibility of representation afforded by fully embracing Semantic Web technologies. By adopting consistent and canonical identifiers for every aspect of chemical entities identified here, we have demonstrated facile cross-domain chemical knowledge integration while preserving correct data correspondence and explicit data provenance information. Furthermore, we have demonstrated the power of CHESS in enabling integrative chemical research that draws on the entirety of chemical information available on the Web. While we do not believe that any specification can natively address outright errors in databases, CHESS representations allow us to explicitly and formally define the meaning of our data and enable machines agents to automatically reason over this data, checking it for consistency and completeness. This, in turn, enables a more accurate scientific discourse and a more reproducible and transparent way of doing science.

We have also demonstrated mechanisms by which chemical configuration-specific information may be encoded without loss of inter-configuration information aggregation and without introducing intra-configuration information mixing that is sometimes an unfortunate occurrence in traditional databases. For example, atomic coordinate information and heat of formation data may exist for multiple conformers of a single molecule, with every conformer annotated with the appropriate data. In principle, this chemical configuration concept allows one to aggregate information about the various electronic states of a given molecule when CHESS will be extended to include the explicit specification of electrons and related concepts. While CHESS does not aim to make statements with respect to the preferred chemical configuration of a given compound under a given set of conditions, CHESS allows the unambiguous and explicit identification of precise chemical configurations for the purpose of advancing and facilitating interdisciplinary scientific discourse.

We believe that outsourcing of chemical information integration to machine agents is of increasing importance as the rapidly growing collection of diverse chemical information already available on the web is overwhelming human integrative capacity. If no steps are taken to create, standardize, and adopt a set of consistent standard Semantic Web chemical information exchange ontologies and representation formalisms soon, we are risking missing yet another opportunity to truly federate the chemical web and trigger a transition to a new era of chemical research. Therefore, with this work, we would like to invite the broader cheminformatics community to initiate the discussion of representations, standards, and supporting ontologies. Truly, the infinite chemical space is full of mysteries, marvels, and opportunities - and we believe that it is only through the concerted and unified efforts of researchers in all fields of science, enabled by Semantic Web technologies, that we may hope to one day chart it.

## Methods

### Supporting Ontologies

CHEMINF is a collaboratively developed Web Ontology Language (OWL) [37] ontology for representing chemical information and descriptors, freely available to the broad cheminformatics community. Semanticscience Integrated Ontology (SIO) is an general ontology that provides over a 150 object relations and over 900 classes of various entities, including physical, processual, and informational ones. There is only one data property in the ontology, 'has value', with the other relations describing aspects of mereology, spatial positioning, temporal ordering, qualities, attributes, representations, participation, and agency. In addition to these ontologies, we use the CHEBI ontology for chemical entities and concepts.

### Triplification of Chemical Information

The encoding of chemical information into its CHESS form was carried out with software we developed (sample source code available on the companion website [23]), based on the Jena API [38] and the Chemistry Development Kit [39]. A sample dataset of 90 antidepressants was obtained from a mesh-based keyword search on 'antidepressant' over the PubChem database. Chemical descriptors were computed using CDK and the Open Babel Java API [40]. Unique identifiers were obtained by hashing pertinent chemical data as a 40-letter SHA-1 hash using the Java Security API.

### Query Testing

In order to test our chemical similarity queries, we used OpenLink Virtuoso version 6.01.3126 SPARQL endpoint [41]. To test DL Rule-based queries, we used the Pellet reasoner version 2.2 [42], running on a single CPU core. The machine used for these tests had dual Intel Xeon CPU at 2.9GHz, with 32GB of RAM.

## Competing interests

The authors declare that they have no competing interests.

## Authors' contributions

LLC wrote the paper, implemented supporting tools, generated triple stores, and ran tests. LLC and MD created the supporting ontologies. MD contributed to the paper, provided guidance, and ran tests. Both authors have read and approved the final manuscript.
